# Recent Developments in Cancer Systems Biology: Lessons Learned and Future Directions

**DOI:** 10.3390/jpm11040271

**Published:** 2021-04-04

**Authors:** Kazim Y. Arga, Raghu Sinha

**Affiliations:** 1 Department of Bioengineering, Marmara University, Istanbul 34722, Turkey; 2 Department of Biochemistry and Molecular Biology, Penn State College of Medicine, Hershey, PA 17033, USA

Cancer is a complex disease involving multiple mechanisms and critical players, at broad genomic, transcriptional, translational and/or biochemical levels. One could envision discovering new biomarkers for early detection by understanding the behavior of cancer development and progression, but to date, there are few biomarkers approved for use in the clinical setting. Therefore, there is a critical need to improve strategies and methods by using novel state-of-the-art tools and strategies to identify and validate newer biomarkers. In addition to biomarkers, there is also a demand for effective methods to identify new targets to inhibit tumor growth. Technically, there is a growing requirement to find new targets using personalized approaches in a targeted and much more effective manner, as existing drugs often become resistant over time in cancer patients. Opportunities to improve this strategy would, therefore, be to find better druggable targets and provide options for drug combinations and/or drug repurposing. More importantly, the ultimate goal of an oncologist and the desire of the cancer patient is to improve overall survival and this could be achieved in part through better prognostic models. Cancer systems biology has undoubtedly emerged as an integrative tool to achieve such advances.

This Special Issue on “recent developments in cancer systems biology” has compiled several novel approaches that use cutting-edge technologies to build a strong foundation of systems biology in cancer research. The issue includes eight original research articles and four literature reviews on recent efforts that use a variety of in silico tools along with experimental approaches to discover novel biomarker candidates for diagnosis and prognosis and to identify drugs and their targets for treatments that could be used in thirteen cancers and their subtypes.

Several “omics” investigations, including genomics, proteomics, metabolomics, glycomics and metagenomics, provide potential candidate biomarkers that can be measured in plasma, tissue and saliva in several lethal cancer types including Pancreatic Cancer [1]. Integrative analysis of these “omics” data would likely discover novel biomarkers for diagnosis and prognosis as well as targets for effective therapy. Moreover, distinguishing clinically similar cancers can be challenging and focusing on genomic and transcriptomic variations may prove beneficial, this issue describes details on various methods available for ovarian and breast cancers [2] and types of lung cancer [3,4] and renal cell carcinoma [5] for identifying key genes and pathways that might assist in proposing diagnostic and prognostic predictions. In addition, integrating multi-omics is important particularly in the use of patient-derived experimental models [6] that can be used in the clinical setting to provide personalized treatment options. Another genome-level advancement that surpasses next-generation sequencing is the identification of somatic structural variants (SVs) that influence functional and cancer-related genes [7]. This optical genome mapping and SVs analysis can be applied to a variety of solid tumors for better cancer prognosis and treatment.

Discovering new targets in cancers provide opportunities especially for recurrences since the drug resistance is proving to be challenging to treat. Several drug targets have been identified using transcriptomics and biological networks in different cancer types including miR-1246 targeting several genes [4] and Sestrin-2 [8] in lung adenocarcinomas, ELK1 [9] and ETS [10] genes in glioma. Additionally, drug repurposing strategies are not only extensively used to discover new uses for already approved drugs, but also provide opportunities for potentially treatment of drug resistance in various cancers. In another article [11], drug repurposing efforts were reviewed in triple-negative breast cancer, an aggressive breast cancer subtype that has a high rate of recurrence and metastasis. These authors compared different repurposing strategies, including structure-based, transcription signature-based, biological network-based and data mining-based drug repositioning. In another study, seven distinct gene programs representing different biological processes involved in drug-induced changes in AML were identified [12]. Furthermore, a data-driven dynamic model of acquired resistance to combined drugs was constructed by these authors and revealed several interventions that can specifically disrupt portions of the system-wide drug response, which could allow co-targeting and lead to synergistic treatments that can overcome resistance and prevent potential recurrence. 

In conclusion, all of the articles published in this Special Issue cover recent developments with attractive approaches to a wide range of topics encompassing the Cancer Systems Biology. These articles and reviews propose a variety of biomarkers for clinical diagnosis, prognosis and therapeutic strategies including “drug repurposing“ for various cancers that pose a major health challenge with significant socioeconomic consequences. We would like to make an appeal to researchers around the world to join forces and contribute to the development of a common platform for personalized medicine using a combination of the different biomarkers proposed in this Special Issue in a diagnostic and/or prognostic setting, allowing the identification of patients at risk, which would facilitate the early initiation of personalized treatments. This Special Issue also highlights the various predictive models and the use of integrated biological network analysis to identify target genes and correlate them with prognosis. It is of utmost importance that all predictive models must undergo extensive validation.
